# Evolutionary dynamics and transmission patterns of Newcastle disease virus in China through Bayesian phylogeographical analysis

**DOI:** 10.1371/journal.pone.0239809

**Published:** 2020-09-29

**Authors:** Jiteng Du, Jing Xia, Shuyun Li, Yuxi Shen, Wen Chen, Yuwen Luo, Qin Zhao, Yiping Wen, Rui Wu, Qigui Yan, Xiaobo Huang, Sanjie Cao, Xinfeng Han, Min Cui, Yong Huang

**Affiliations:** Key Laboratory of Animal Disease and Human Health of Sichuan Province, College of Veterinary Medicine, Sichuan Agricultural University, Chengdu, Sichuan, People's Republic of China; Beni Suef University, Faculty of Veterinary Medicine, EGYPT

## Abstract

The Chinese poultry industry has experienced outbreaks of Newcastle disease (ND) dating back to the 1920s. However, the epidemic has exhibited a downtrend in recent years. In this study, both observational and genetic data [fusion (F) and haemagglutinin-neuraminidase genes (HN)] were analyzed, and phylogeographic analysis based on prevalent genotypes of Newcastle disease virus (NDV) was conducted for better understanding of the evolution and spatiotemporal dynamics of ND in China. In line with the observed trend of epidemic outbreaks, the effective population size of F and HN genes of circulating NDV is no longer growing since 2000, which is supported by 95% highest posterior diversity (HPD) intervals. Phylogeographic analysis indicated that the two eastern coastal provinces, Shandong and Jiangsu were the most relevant hubs for NDV migration, and the geographical regions with active NDV diffusion seemed to be constrained to southern and eastern China. The live poultry trade may play an important role in viral spread. Interestingly, no migration links from wild birds to poultry received Bayes factor support (BF > 3), while the migration links from poultry to wild birds accounted for 64% in all effective migrations. This may indicate that the sporadic cases of ND in wild bird likely spillover events from poultry. These findings contribute to predictive models of NDV transmission, and potentially help in the prevention of future outbreaks.

## Introduction

Newcastle disease (ND) is one of the most contagious diseases of poultry. The causative agent of ND is known as Newcastle disease virus (NDV), which is a member of the family *Paramyxoviridae* in the genus *Avulavirus* [1, 2]. It was first reported both in Java, Indonesia and Newcastle-upon-Tyne, England in 1926. Since then, four epidemic waves have occurred worldwide through the 1990s [3]. In China, the time of the first probable ND outbreak was nearly synchronous with the initial global epidemic in the 1920s. It was not until 1946 that the etiology of the outbreak in China was identified. This delay resulted in enormous losses to the poultry industry. Although ND poses a threat to the Chinese chicken industry, the number of outbreaks, cases and deaths since 2005–2015 have been decreasing year by year due to a strict vaccination program [4]. Most cases that occurred were mild and sporadic in Chinese vaccinated chicken flocks, which may be the result of immune failure [5]. Characteristic of “atypical ND”, presents as a prolonged disease duration with no typical clinical and pathological manifestation [6]. Similar to other single-stranded-RNA respiratory viruses, multiple genotypes of NDV can co-circulate and cause outbreaks, and the “mild ND” under long-term immune pressure may also provide the conditions for the evolution of the virus.

In general, the capacity of some viruses to adapt to hosts and environments is highly dependent on their ability to generate de novo diversity in a short period of time [7], and prevalent genotypes of viruses tend to have a higher evolutionary rate under more selection pressure [8]. Low evolutionary rates of fusion (F) gene exhibited in genotypes II and IX of virulent NDV (7.05 × 10^−5^ and 2.05 × 10^−5^ per year, respectively) make that there is a high genetic similarity to virulent isolates from the 1940s [9], while the evolutionary rate and diversity of the predominant NDV genotypes VI and VII in China remain unknown. According to Fan (2017), nucleocapsid protein is observed with an unexpected rapid evolutionary rate, 1.059 × 10^−2^ per year (95% HPD: 4.187 × 10^−3^ ~ 1.74 × 10^−2^) rather than surface proteins (F and HN) of NDV [10]. Wrong estimation of the evolutionary rate of viruses may significantly affect the prevention and control of viral diseases [11, 12].

Similar with the avian influenza virus, wild birds are also considered to play an important role in the spread of ND by the high nucleotide homology of viruses between wild birds and poultry [13–15]. However, the directionality of viral transmission between wild birds and poultry remains unknown.

The present study aimed to estimate the evolutionary rate and diversity of surface protein of the predominant NDV genotypes in China, and explore the evolutionary dynamics and transmission patterns in multiple hosts of NDV in China using phylodynamics analysis.

## Materials and methods

### Epidemiologic data

Clinical case data of NDV were obtained from the *Official Veterinary Bulletin*, which is made available by the Ministry of Agriculture and Rural Affairs of the People’s Republic of China [16]. We collected the data (S1 Table), including the total number of outbreaks, number of cases and deaths, province and animal species in 2006–2019 by month and compared the distribution of NDV outbreaks in China.

### Sequence data

All NDV sequences used in this study were obtained from GenBank, which were sampled in China between 1985 and 2015. The F and HN gene sequences were screened by filtering out those that were duplicate and incompletely or vaguely annotated. The screened and reference sequences were aligned using the Clustal W method in MEGA7 [17] to perform genotyping.

Recombinant sequences were detected using RDP 4.95 [18]. TempEst1.5.1 [19] was used to examine the temporal signal and look for problematic sequences. Furthermore, three random down-samples were created with a maximum of 15 sequences of VII-F gene per location to assess the robustness of the phylogeographic reconstructions against sampling biases. As only small number (less than 150 taxa) of VI-F and VII-HN gene sequences are available in GenBank, they were retained with no random down-sampling for the following analysis. The alignment sequence data of NDV used for analysis are listed in S1 Dataset.

### Phylogenetic analysis

Time-scaled phylogenetic trees of NDV were reconstructed using a Bayesian inference approach by Bayesian Evolutionary Analysis Sampling Trees (BEAST) (model selection: BEAST v2.5.1; analysis: BEAST v1.10.4) with BEAGLE [20–22]. All analyses were performed using the GTR+I+Γ4 nucleotide substitution model by jModelTest v2.1.7 [23]. Relaxed clock [24] with uncorrelated lognormal distribution (UCLD), relaxed clock with uncorrelated exponential distribution (UCED), Strict clock and Tree priors (Coalescent Constant Size, Coalescent Exponential Growth and Coalescent Bayesian Skyline) were combined in different combinations to calculate their respective marginal L estimate values by Path sampling [25] (Nr of Steps: 100; Chain Length: 100,000; Pre Burnin: 10,000) to find best-fit model in BEAST v2.5.1. UCED relaxed clock with Bayesian skyline model was best-fit for all genotype subsets (S2 Table). A Markov Chain Monte Carlo (MCMC) chain of 100 million with sub-sampling every 10,000 generations was specified. Convergence was assessed by estimating the effective sampling size (ESS) after a 10% burn-in using Tracer v1.7 [26]. The ESS was over 200 for parameter estimation in the MCMC analysis. Maximum clade credibility (MCC) trees were summarized in Tree Annotator v1.75 [27] and visualized using FigTree v1.4.3 [28].

The Coalescent Bayesian skyline plot (BSP) was used to infer the past population dynamics [29]. The uniform sampling strategies were used to select datasets with a maximum of 20 sequences per year [30]. To avoid the effect of left censoring [31], the BSPs were truncated at the time of the last coalescent event. Package Tracer was used to plot BSP and lineages-through-time (LTT) plots.

### Bayesian phylogeography analysis

An asymmetric discrete trait phylogeography model was specified to explore the spatial diffusion patterns of NDV. Both location and hosts were imported into the model to infer a spreading network with Bayesian stochastic search variable selection (BSSVS) using BEAST 1.10.4 [32]. SpreaD3 v0.9.6 was used to calculate Bayes factor support for each transmission path between discrete location states and hosts [33]. The settings used here can be found in the SpreaD3 tutorials [34]. Only migration links with Bayes factor support of at least 3 were considered. Also, the number of expected location-state and host-state transitions (Markov jump counts) along the branches of the phylogeny using the asymmetric migration model were estimated [35]. Total number of state counts for migration into and out of each region and host were also plotted.

To uncover potential predictors of viral spread, we tested the association between the viral dispersal and predictors (including environmental predictors, poultry farming and live poultry trade predictors) among provinces using generalized linear model (GLM). GLM analyses were run in BEAST v1.10 using prior specifications recommended above on the set of trees obtained by Bayesian phylogenetic analysis [36, 37]. The province-level matrix data of live poultry transportation were referred to a recent research [38]. Province-level poultry farming data (including domestic broiler/layer population of each large, medium and small scale chicken farms and annual output of poultry) were obtained from statistical yearbooks of China, and annual relative humidity and temperature data were obtained from China Meteorological Administration (S3 Table).

## Results

### Epidemiology

**Fig 1:**
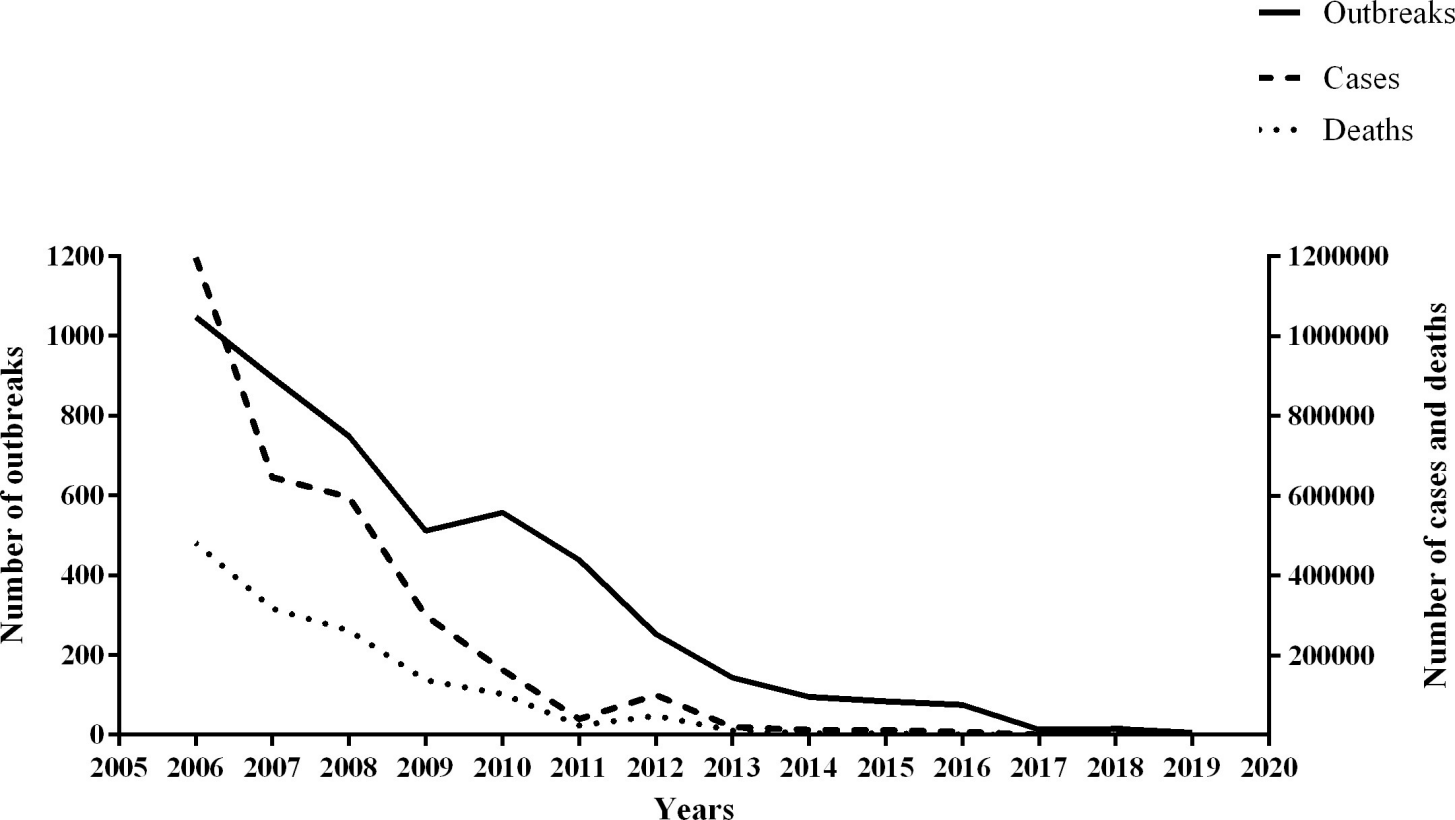
The total number of annual outbreaks, cases, and deaths of NDV in China from 2006 to 2019.

From 2006 to 2019, a total of 4,789 ND epidemics were reported in China, covering 26 provinces, municipalities and autonomous regions, and the Chinese epidemics of ND primarily occurred in the south and southwest of China (S1 Fig). The number of NDV outbreaks showed a downtrend each year, as did the number of cases and deaths over time (Fig 1).

### Sequence dataset compilation

The F (N = 876) and HN (N = 387) gene sequences were downloaded from GenBank, and only sequences subtyped as VI and VII-type were retained (F gene: N = 753; HN gene: N = 177). The duplicate, problematic sequences and short (nucleotide length < 1,600 base pair) sequences were removed, leaving 444 taxa of VII-F gene, 72 taxa of VI-F gene, 132 taxa of VII-HN gene and 18 taxa of VI-HN gene. And the VI-HN gene sequences were not used for the subsequent study due to its small number.

NDV infection was reported in 19 host species so far. Of these, 6.25% of the VI and VII genotype viruses were reported in wild species and more in domestic poultry (93.56%). Among 25 discrete regions, most NDVs were isolated in Shandong (25.57%) and Jiangsu (24.81%) provinces, followed by Heilongjiang (7.95%) and Guangdong (7.58%). To mitigate the potential impact of sampling biases in following phylodynamic reconstructions, three randomly down-sampling were used to select datasets with a maximum of 15 taxa per location. After down-sampling randomly, three final sets (N = 177, 171 and 178) of VII-F gene were used in the following analysis. As there was small number of sequences of VI-F gene and VII-HN gene, all of the sequences were retained, and the meta-data was listed in S4 Table.

### Phylogenetic and population dynamic analysis

**Fig 2:**
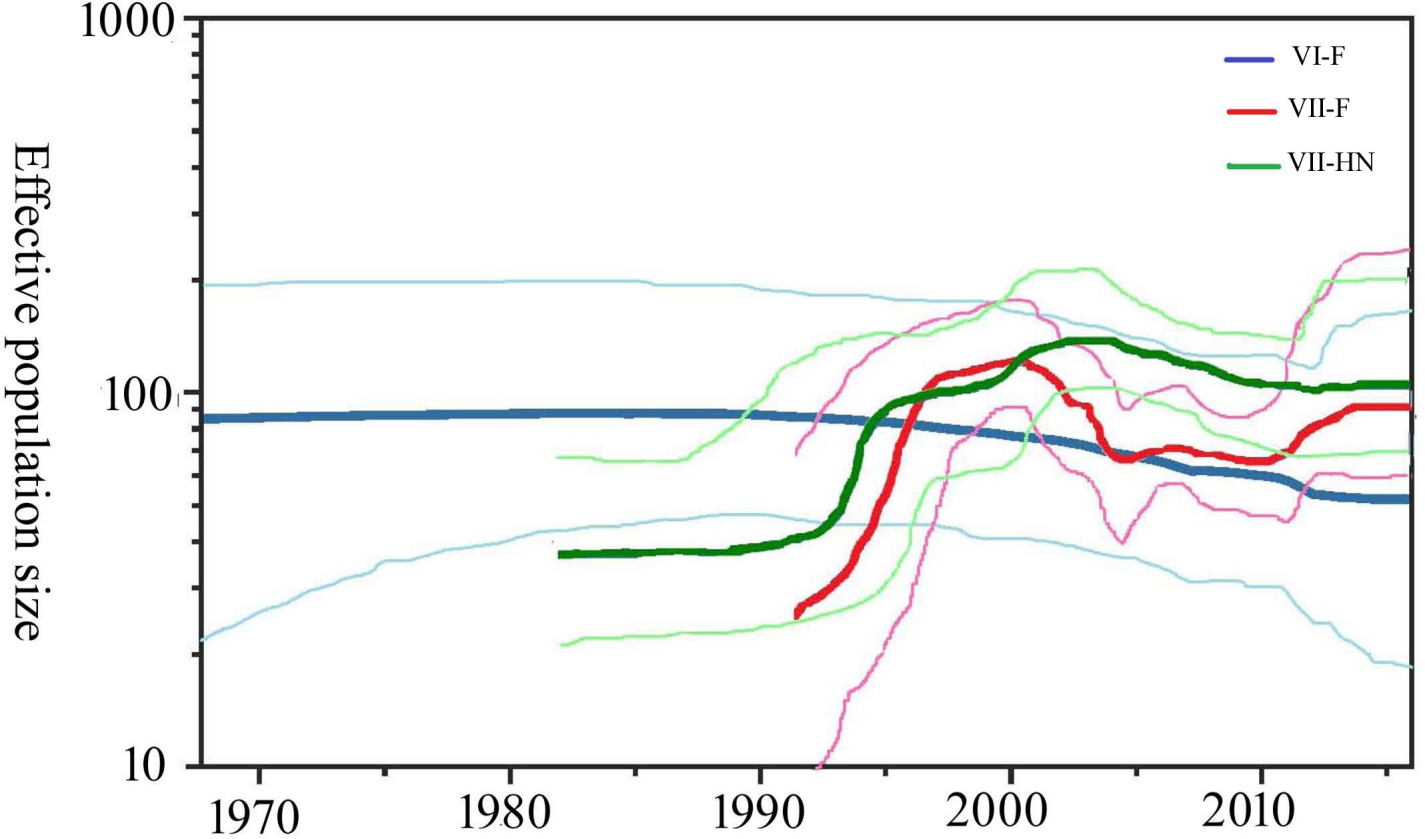
Bayesian skyline plot of genotypes VI and VII NDVs in China. Effective population size estimates are expressed on a logarithmic scale (Y-axis), and the x-axis represents time (years). The dark thick lines denote the median estimates and the light thin lines give the 95% HPD intervals of the estimate. The dark blue, red and green thick lines represent the median estimates of VI-F gene, VII-F gene and VII-HN gene, respectively. The light blue, red and green thin lines represent the 95% HPD intervals of the estimates of VI-F gene, VII-F gene and VII-HN gene, respectively.

A examine for molecular clock signal revealed that there was sufficient accumulation of divergence over the sampling time span to estimate evolutionary rates (S2 Fig). The evolutionary rates and past population dynamics of NDV were inferred using a Bayesian coalescent approach. The mean evolutionary rates of the VI-F, VII-F and VII-HN genes were estimated at 8.07 × 10^−4^ subs/site/year (95% HPD: 5.06 × 10^−4^ ~ 1.09 × 10^−3^), 1.03 × 10^−3^ subs/site/year (95% HPD: 8.54 × 10^− 4^ ~ 1.19 × 10^−3^) and 8.78 × 10^−4^ subs/site/year (95% HPD: 7.11 × 10^−4^ ~ 1.05 × 10^−3^), respectively. For the effective population size of three subsets (VI-F, VII-F and VII-HN), the LTT graphs (S3 Fig) showed that there were no new lineages since 2013. Therefore, we assumed that there was no change imputed in the effective population size from 2013 onwards. Effective population size in BSP plots of VII-F and VII-HN genes showed that an increasing trend was observed from 1995 to 2000, and the trend from 2000 to 2013 kept relatively constant (Fig 2). Compared with VII-genotype, the effective population size of VI-F gene was relatively stable since the 1970s supported by a 95% HPD interval (Fig 2).

The MCC tree showed (S4 Fig) that the most recent common ancestor (MRCA) for the VI-F gene was estimated to be 1934 (95% HPD: 1897 ~ 1972), which might originate in Southeastern China (Jiangxi province). The majority of VII genotype viruses which fell into four phylogenetic clades (VIId, VIIc, VIIe and VIIf) might originate in Guangxi province. MRCA estimation of both F and HN gene was similar with approximately 1984 (95% HPD: 1975 ~ 1988) and 1972 (95% HPD: 1962~ 1978), respectively. Sub-genotype VIId viruses of VII-NDVs were the majority clades since 2008, and a small group of two new clades VIIe- and VIIf-NDVs were isolated since 2012.

The MCC tree based on hosts (S4D Fig) demonstrated that the earliest genotype VI NDVs in China might be derived from pigeons. The MCC trees inferred from F and HN genes based on hosts (S4E and S4F Fig) showed that the earliest source of genotype VII virus seemed to be chicken and subsequently spread to other poultry and wild birds.

### Phylogeographic analysis

**Fig 3:**
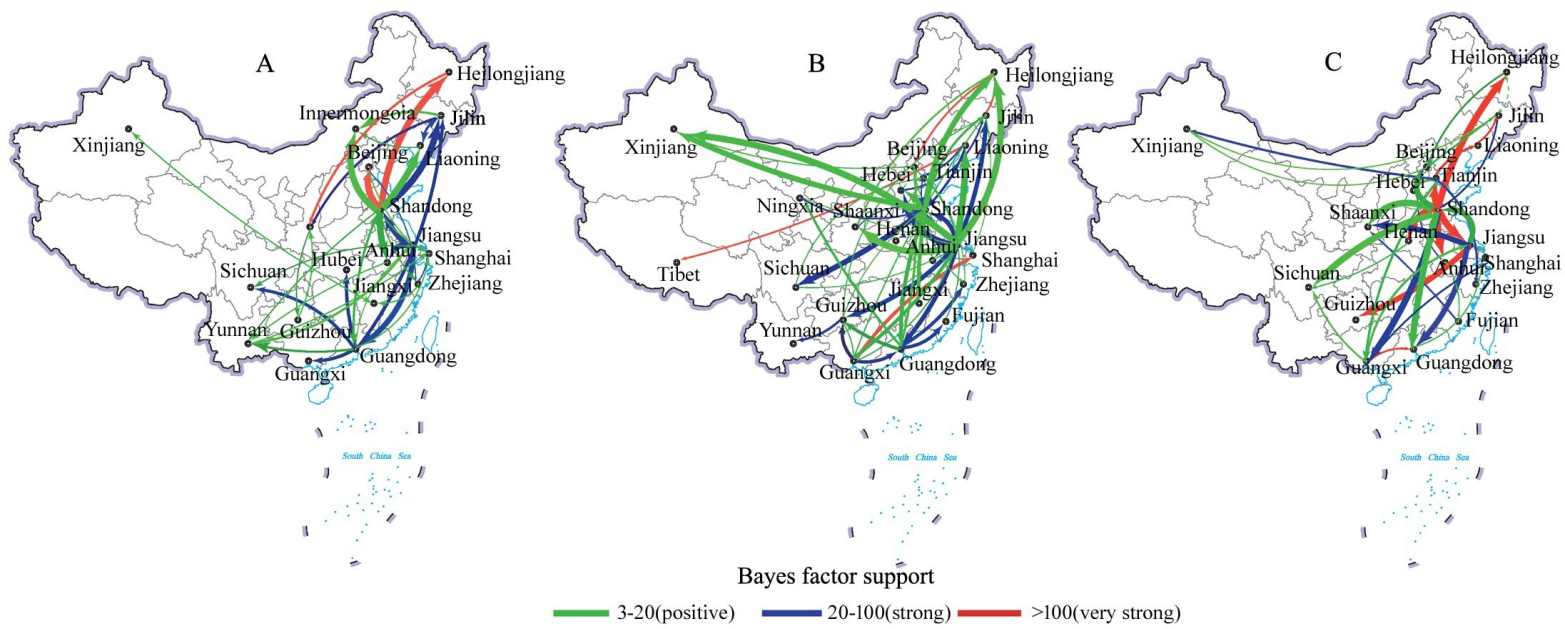
Migration link map of genotypes VI and VII NDVs in China supported by Bayes factor. **(**A) VI-F gene, (B) VII-F gene (subsample one), (C) VII-HN gene. The line colour represents the relative strength by which the rates are supported: very strong (BF > 100, red lines), strong (20 < BF < 100, blue lines) and positive (3 < BF < 20, green lines). The thickness of the arrows indicates increasing number of Markov jumps between locations.

Among 25 provinces, municipalities, and autonomous territories of China, a total of 285 migration links of well supported (Bayes factor support, BF > 3) were identified for VI-F, VII-F and VII-HN genes (S5 Table). Herein, the total number of 156 migration links were the sum of VI-F, VII-F (subsample one) and VII-HN BSSVS outputs (Fig 3). The eastern seaboard of China, Shandong (29.50%; N = 46/156) and Jiangsu (21.20%; N = 33/156) provinces were the most frequently implicated source and recipient location, followed by southern seaboard of China, Guangdong (16.02%; N = 25/156) and Guangxi (12.80%; N = 20/156). The results showed that the eastern seaboard of Shandong and Jiangsu provinces might have played a key role in seeding the NDV epidemics. This is further supported by the number of observed state changes in Markov jump count analysis with migration into and out of Shandong and Jiangsu provinces, which was higher than any other region (S5 Fig). Furthermore, the visual migration maps (Fig 3) indicated that the eastern and southern regions of China seemed to become the hot spots of NDV diffusion.

The data sets of VI-F, VII-F and VII-HN genes were used to infer bird migration history and the Bayes factor support for each migration path between hosts were estimated using BSSVS. Between the 19 hosts (poultry: 5, wild birds: 14) identified by F and HN genes, 44 routes (counting strategy is the same as above) of statistically supported (BF > 3) were identified. Interestingly, among all supported well migration paths (S6 Table), the migration directions were spread from poultry to wild birds accounted for 58.10% (N = 25/44), between poultry accounted for 30.30% (N = 13/44), and between wild birds accounted for 11.60% (N = 5/44). However, no migration links of wild birds to poultry were observed. This observation indicates that the sporadic cases of ND in wild birds are likely spillover from poultry. Besides, the number of expected host-state migrations was also estimated in this study. Pigeons may play an important role in the transmission of NDV genotype Ⅵ, with the largest into and output sources of virus. According to the outcomes of Markov jump counts analysis based on F and HN genes of VII genotype (S6 Fig). Chicken is the biggest output source of VII-NDVs, which spread to other poultry/wild birds, such as duck and goose. These results demonstrated that NDV appears to mainly spread from poultry to other poultry and wild birds.

**Fig 4:**
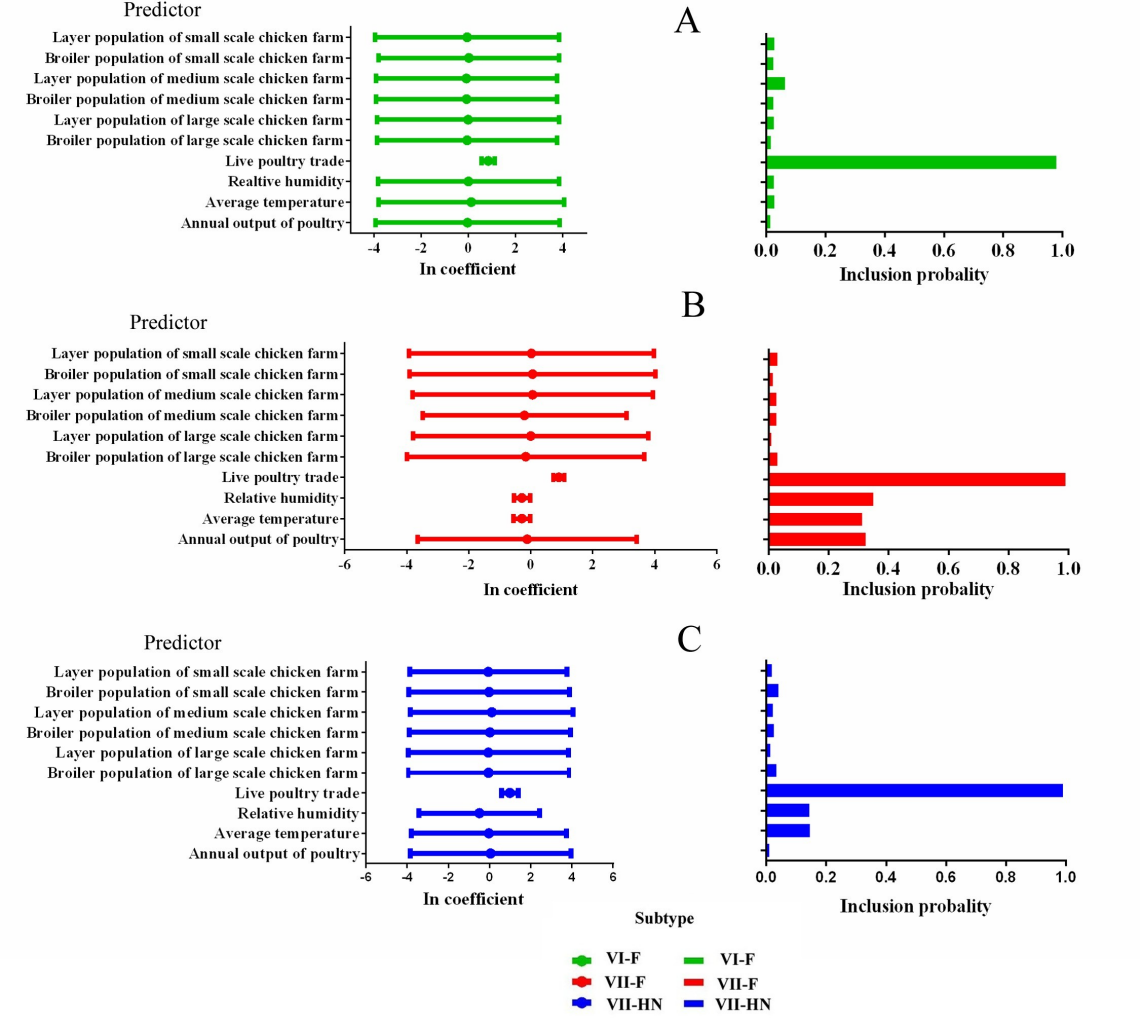
Predictors of NDV dispersal across China. (A), (B) and (C) represent the contributions of predictor variables to the dissemination of viral VI-F, VII-F and VII-HN genes, respectively; VI-F,VII-F and VII-HN genes are shown as green, red and blue, respectively. (Left) Circles show the estimated conditional effect sizes for the predictor coefficients (> 0 = positive association, < 0 = negative association). Error bars represent the 95% highest posterior density (HPD) credible interval for these estimates. (Right) Bars show the posterior probability of inclusion of each predictor in the model.

The results GLM analysis inferred from the data sets of VI-F, VII-F and VII-HN genes showed that live poultry trade network is positively associated with viral spread (Fig 4). In addition to the predictor of live poultry trade, other biological potential predictors and abiotic predictors had also been estimated, but they did not get noticeable support by any of the analyzed datasets (Fig 4).

## Discussion

In the present study, large amounts of epidemiologic and genetic data, along with associated temporal and geographic information, were collected to investigate the emergence and dispersal of the predominant genotypes VI and VII NDV in China. The number of outbreaks, cases, and deaths of NDV in China has been decreasing in recent years. A strict immunization program in conjunction with a reduction in the numbers of backyard poultry are the major contributors to the decline of ND in domestic poultry. Phylogeographic analysis based on prevalent genotypes of NDV was conducted for the better understanding of the evolution and spatiotemporal dynamics of ND in China.

Bayesian coalescent analysis revealed the mean substitution rates of F gene of genotypes VI and VII and HN gene of genotype VII were 8.07 × 10^−4^ subs/site/year (95% HPD: 5.06 × 10^−4^ ~ 1.09 × 10^−3^), 1.03 × 10^−3^ subs/site/year (95% HPD: 8.54 × 10^−4^ ~ 1.19 × 10^−3^) and 8.78 × 10^−4^ subs/site/year (95% HPD: 7.11 × 10^−4^ ~ 1.05 × 10^−3^), respectively. The evolutionary rates in this study were slightly lower than the rates estimated in a previous study [39] for full-length F and HN genes sequences. However, it still lies within the 95% HPD interval of estimations for F gene (0.71 × 10^−3^ ~ 1.98 × 10^−3^) subs/site/year and (0.51 × 10^−3^ ~ 1.68 × 10^−3^) subs/site/year for HN gene, respectively. The differences in evolutionary rates may be due to the use of sequences only including Chinese isolates in present study. Based on these evolutionary rates, the MRCA was established to be around 1934 (95% HPD: 1897 ~ 1972) for Ⅵ-F gene, which is compatible with the first outbreak record of ND in China in 1935 [3]. Similar to VI-F genes, the MRCA of VII-F and VII-HN genes estimated in the present study is matching with a recent study [39].

In the previous study, the BSP analysis of NP gene performed by Fan [10], suggesting the population size of NDV showed an increase in the 1990s. Similar results were obtained in our study of population dynamics history for NDV genotype Ⅶ, which are summarized in a BSP supported by a narrow 95% HPD (Fig 2). This increase may be closely related to the fourth panzootic of NDV worldwide [40–42]. Genotype Ⅶ virus evolved into epidemic lineages and the viruses spread to most parts in China during this time [43, 44]. Unlike previous studies, the population dynamics observed after 2000s displayed a different behavior since a relatively stable trend in the effective population size was observed. Although the factors responsible for the observed population size are currently unknown, a compulsory vaccination program has been considered to be a major factor leading to the death of some lineages [39]. While, the extant diversity has not decreased over time, the circulating of mutants and/or new sub-genotypes of the virus may keep the stable effective population of NDVs in China [45, 46]. More studies should be carried out to explore these changes.

Two eastern seaboard provinces, Shandong and Jiangsu, were identified as the most frequently implicated source and recipient location, which might play central roles for NDV spread in China. This finding is also supported by the number of observed state changes in Markov jump count analysis with migration into and out of Shandong and Jiangsu provinces, which was greater than any other region (S5 Fig). Visualizing migration links (BF > 3) revealed more detail about the migration patterns of the virus (Fig 3). All these migration maps reflected that the eastern and southern seaboard of China became the active regions in the transmission of NDV. The Bohai Economic Rim, Pearl River Delta and Yangtze River Delta regions, located in eastern and southern China, are the most densely populated and convenient transportation network in China, making them the economic powerhouses of the country [47]. Those regions are also the places with the highest density of poultry farming in China. However, GLM analysis showed that the viral dispersal may not directly associate with the density of poultry. The possible reason is that both broilers and layers are vaccinated with NDV vaccine nationwidely, which causing the low risk of NDV outbreak in most farms. Live poultry transport is presumed to be related to the viral spread. While, how the virus is transmitted in live poultry transporting remains unclear.

Long-distance migratory birds play a major role in the global spread of avian influenza viruses in previous studies [48–50], while the role of wild bird migration in the spatial diffusion of NDV is unknown. In our study, several long migration paths, such as Beijing to Xinjiang, Jiangsu to Tibet, and Guangdong to Shandong were observed, which are associated with NDV migration. Surprisingly, all these routes are associated with spread by poultry (S4 Fig). By counting the migration links (BF > 3) of NDV spread between diverse hosts (S6 Table), no migration links supported by BF (BF > 3) and the direction was from wild birds to poultry. Furthermore, the number of expected host-state migrations (Markov jump counts) estimated in this study also demonstrated that poultry (i.e. Chicken and pigeon) were the main output source of NDV expansion and contributed most to the virus spread (S6 Fig). Therefore, we speculated that the NDV mainly migrated from poultry to poultry/wild birds. However, this result could be affected by the lack of NDV samples from wild birds [51].

A major limitation of any phylodynamic analysis is the dependence on sampling [52]. It is an inherent issue that sampled viruses are concentrated in high-risk areas, potentially resulting in sampling bias and inaccurate ancestral reconstruction processes [53]. Similar to previous studies, an attempt was made to reduce sampling biases by down-sampling with a maximum of 15 sequences per location [54, 55]. However, owing to passive and active surveillance in wild bird populations appears to be very limited for NDV in China, the few available sequences were collected from wild birds. Therefore, we did not opt for a down-sampling to obtain even number of sequences by host category to infer the contribution of wild bird in the diffusion of NDV, which inevitably leads to potential biases and limitations of the results. Furthermore, we recommend that the active systemic surveillance of wild birds should be strengthened and valued to obtain more viral samples from wild birds.

## Conclusion

Our study demonstrates that the number of outbreaks, cases and deaths of NDV appeared to be gradually decreasing since 2006, and a relative stable trend in the effective population size was observed in the predominant genotypes of NDVs in recent ten years. The regions of Shandong and Jiangsu were estimated to be the most relevant hubs for NDV migration, and the live poultry trade may play an important role in viral spread. Also, the potential of NDV migration appeared to be the highest between poultry fowl and spillover from poultry to wild birds. These findings extend our understanding of dispersal patterns of the predominant genotypes of NDV and cross-hosts transmission in China, which may improve awareness and future control capability and other important avian pathogens.

## Supporting information

S1 Fig Spatial distribution of NDV epidemic from 2006 to 2019. Different shades of color represent the total number of outbreaks in the region. The deeper the color, the more outbreaks of NDV in the region.
(TIF)

S2 Fig Plots of the divergence from the root of the tree against time of sampling. The X-axis represents time, Y-axis represents root-to-tip divergence, the line is the best-fit regression. The red spot represents the problematic sequences and has been removed in the phylogenetic and phylogeographic analysis.
(TIF)

S3 Fig Lineages-Through-Time (LTT) plot from BEAST. (A) VI-F gene, (B) VII-F gene, (C) VII-HN gene.
(TIF)

S4 Fig The Maximum Clade Credibility (MCC) trees of NDV. (A), (B) and (C) represent the MCC trees based on locations of VI-F gene, VII-F (subsample 1) and VII-HN genes, respectively; (D), (E) and (F) represent the MCC trees based on hosts of VI-F gene, VII-F (subsample 1) and VII-HN gene respectively. Lines of diverse colors represent different locations or host origins. The scale bar represents the unit of time (year).
(TIF)

S5 Fig Histograms of the total number of location-state transitions. (A) VII-F gene (subsample 1), (B) VII-F gene (subsample 2), (C) VII-F gene (subsample 3), (D) VI-F gene, (E) VII-HN gene.
(TIF)

S6 Fig Histograms of the total number of host-state transitions. (A) VII-F gene (subsample 1), (B) VII-F gene (subsample 2), (C) VII-F gene (subsample 3), (D) VI-F gene, (E) VII-HN gene.
(TIF)

S1 Table NDV epidemic data in China between 2006 and 2019 collected from *Official Veterinary Bulletin*. (XLSX)

S2 Table Best model screening of NDV. (DOCX)

S3 Table The matrix data of environmental predictors, poultry farming and live poultry trade predictors used for GLM analysis. (XLSX)

S4 Table Summary of F and HN genes sequence data of NDV by collected location, host and year from 1986 to 2015 in China, and the accession numbers of the sequences used for analysis. (XLSX)

S5 Table Posterior probabilities and Bayes factor support for diffusion between discrete locations of NDV. (DOCX)

S6 Table Posterior probabilities and Bayes factor support for diffusion between diverse hosts of NDV. (DOCX)

S1 Dataset The alignment sequence data of NDV used for analysis. (ZIP)

## References

[1] Mayo MA. A summary of taxonomic changes recently approved by ICTV. 147(8):1655–6. 10.1007/s007050200039 12181683

[2] Mayo MA. Virus Taxonomy—Houston 2002. Archives of Virology. 2002;147(5):1071–6. 10.1007/s007050200036 12021875

[3] Liu H, Wang Z, Wang Y. The History and Status of Newcastle Disease. China Animal Health Inspection 2015;6.

[4] Zhang P, Xie G, Liu X, Ai L, Chen Y, Meng X, et al High Genetic Diversity of Newcastle Disease Virus in Wild and Domestic Birds in Northeastern China from 2013 to 2015 Reveals Potential Epidemic Trends. Applied and environmental microbiology. 2015;82(5):1530–6. Epub 2015/12/30. 10.1128/AEM.03402-15 26712543 PMC4771317

[5] Umali DV, Ito H, Shirota K, Ito T, Katoh H. Atypical velogenic Newcastle disease in a commercial layer flock in Japan. Poult Sci. 2015;94(5):890–7. 10.3382/ps/pev011 . 25810410

[6] Zhang S, Xiaoting W, Changguang Z, Dehua L, Yanxin H, Jixun Z, et al Phylogenetic and Pathotypical Analysis of Two Virulent Newcastle Disease Viruses Isolated from Domestic Ducks in China. Plos One. 6(9):e25000–. 10.1371/journal.pone.0025000 21949828 PMC3176290

[7] Sanjuan R, Domingo-Calap P. Mechanisms of viral mutation. Cell Mol Life Sci. 2016;73(23):4433–48. Epub 2016/10/23. 10.1007/s00018-016-2299-6 27392606 PMC5075021

[8] Brayne AB, Dearlove BL, Lester JS, Kosakovsky Pond SL, Frost SDW. Genotype-Specific Evolution of Hepatitis E Virus. J Virol. 2017;91(9). Epub 2017/02/17. 10.1128/JVI.02241-16 28202767 PMC5391457

[9] Dimitrov KM, Lee DH, Williams-Coplin D, Olivier TL, Miller PJ, Afonso CL. Newcastle Disease Viruses Causing Recent Outbreaks Worldwide Show Unexpectedly High Genetic Similarity with Historical Virulent Isolates from the 1940's. Journal of Clinical Microbiology. 2016;54(5):1228 10.1128/JCM.03044-15 26888902 PMC4844730

[10] Fan W, Xu Y, Zhang P, Chen P, Zhu Y, Cheng Z, et al Analysis of molecular evolution of nucleocapsid protein in Newcastle disease virus. Oncotarget. 2017;8(57):97127–36. 10.18632/oncotarget.21373 29228598 PMC5722550

[11] Worobey M, Han GZ, Rambaut A. A synchronized global sweep of the internal genes of modern avian influenza virus. Nature. 2014;508(7495):254–7. Epub 2014/02/18. 10.1038/nature13016 24531761 PMC4098125

[12] Vrancken B, Suchard MA, Lemey P. Accurate quantification of within- and between-host HBV evolutionary rates requires explicit transmission chain modelling. Virus Evol. 2017;3(2):vex028. Epub 2017/10/14. 10.1093/ve/vex028 29026650 PMC5632516

[13] Schuler KL, Justice-Allen AE, Jaffe R, Cunningham M, Thomas NJ, Spalding MG, et al Expansion of an Exotic Species and Concomitant Disease Outbreaks: Pigeon Paramyxovirus in Free-Ranging Eurasian Collared Doves. Ecohealth. 2012;9(2):163–70. 10.1007/s10393-012-0758-6 22476688

[14] Xiang B, Han L, Gao P, You R, Wang F, Xiao J, et al Spillover of Newcastle disease viruses from poultry to wild birds in Guangdong province, southern China. Infection Genetics & Evolution. 2017;55:199–204. 10.1016/j.meegid.2017.09.020 28935610

[15] Ayala AJ, Dimitrov KM, Becker CR, Goraichuk IV, Arns CW, Bolotin VI, et al Presence of Vaccine-Derived Newcastle Disease Viruses in Wild Birds. PLoS One. 2016;11(9):e0162484 Epub 2016/09/15. 10.1371/journal.pone.0162484 27626272 PMC5023329

[16] Official Veterinary Bulletin. The Ministry of Agriculture and Rural Affairs of the People’s Republic of China. [Cited 2019 Sep 3]:available at http://www.moa.gov.cn/gk/sygb/.

[17] Kumar S, Stecher G, Tamura K. MEGA7: Molecular Evolutionary Genetics Analysis Version 7.0 for Bigger Datasets. Molecular Biology & Evolution. 2016;33(7):1870. 27004904 10.1093/molbev/msw054 PMC8210823

[18] Martin DP, Murrell B, Golden M, Khoosal A, Muhire B. RDP4: detection and analysis of recombination patterns in virus genomes. Virus Evol. 2015;1(1):vev003 10.1093/ve/vev003 27774277 PMC5014473

[19] Rambaut A, Lam TT, Max Carvalho L, Pybus OG. Exploring the temporal structure of heterochronous sequences using TempEst (formerly Path-O-Gen). Virus Evol. 2016;2(1):vew007 10.1093/ve/vew007 27774300 PMC4989882

[20] Suchard MA, Rambaut A. Many-core algorithms for statistical phylogenetics. Bioinformatics. 2009;25(11):1370–6. 10.1093/bioinformatics/btp244 19369496 PMC2682525

[21] Bouckaert R, Vaughan TG, Barido-Sottani J, Duchene S, Fourment M, Gavryushkina A, et al BEAST 2.5: An advanced software platform for Bayesian evolutionary analysis. PLoS Comput Biol. 2019;15(4):e1006650 Epub 2019/04/09. 10.1371/journal.pcbi.1006650 30958812 PMC6472827

[22] Suchard MA, Lemey P, Baele G, Ayres DL, Drummond AJ, Rambaut A. Bayesian phylogenetic and phylodynamic data integration using BEAST 1.10. Virus Evol. 2018;4(1):vey016 10.1093/ve/vey016 29942656 PMC6007674

[23] Posada D. Selection of models of DNA evolution with jModelTest. Methods in Molecular Biology. 2009;537:93 10.1007/978-1-59745-251-9_5 19378141

[24] Ho SY, Phillips MJ, Drummond AJ, Cooper A. Accuracy of rate estimation using relaxed-clock models with a critical focus on the early metazoan radiation. Mol Biol Evol. 2005;22(5):1355–63. Epub 2005/03/11. 10.1093/molbev/msi125 . 15758207

[25] Baele G, Lemey P, Bedford T, Rambaut A, Suchard MA, Alekseyenko AV. Improving the accuracy of demographic and molecular clock model comparison while accommodating phylogenetic uncertainty. Mol Biol Evol. 2012;29(9):2157–67. 10.1093/molbev/mss084 22403239 PMC3424409

[26] Rambaut A, Drummond AJ, Dong X, Baele G, Suchard MA. Posterior Summarization in Bayesian Phylogenetics Using Tracer 1.7. Systematic Biology. 2018;67(5). 10.1093/sysbio/syy032 PMC6101584 29718447

[27] Rambaut A, Drummond AJ. TreeAnnotator v1.5.3: MCMC Output Analysis. 2018;available at http://beast.bio.ed.ac.uk/TreeAnnotator.

[28] Rambaut A, Drummond AJ. FigTree 1.4.3. (2016):available at http://tree.bio.ed.ac.uk/software/figtree/.

[29] Drummond AJ, Rambaut A, Shapiro B, Pybus OG. Bayesian coalescent inference of past population dynamics from molecular sequences. Mol Biol Evol. 2005;22(5):1185–92. 10.1093/molbev/msi103 . 15703244

[30] Hall MD, Woolhouse ME, Rambaut A. The effects of sampling strategy on the quality of reconstruction of viral population dynamics using Bayesian skyline family coalescent methods: A simulation study. Virus Evol. 2016;2(1):vew003 10.1093/ve/vew003 27774296 PMC4989886

[31] de Silva E, Ferguson NM, Fraser C. Inferring pandemic growth rates from sequence data. J R Soc Interface. 2012;9(73):1797–808. 10.1098/rsif.2011.0850 22337627 PMC3385754

[32] Lemey P, Rambaut A, Drummond AJ, Suchard MA. Bayesian phylogeography finds its roots. Plos Computational Biology. 2009;5(9):e1000520 10.1371/journal.pcbi.1000520 19779555 PMC2740835

[33] Bielejec F, Baele G, Vrancken B, Suchard MA, Rambaut A, Lemey P. SpreaD3: Interactive Visualization of Spatiotemporal History and Trait Evolutionary Processes. Molecular Biology & Evolution. 2016;33(8):2167–9. 27189542 10.1093/molbev/msw082 PMC6398721

[34] Filip Bielejec GB, Bram Vrancken,Marc A. Suchard,Andrew Rambaut,Philippe Lemey. SpreaD3: Spatial Phylogenetic Reconstruction of Evolutionary Dynamics using Data-Driven Documents (D3) 2016;available at https://rega.kuleuven.be/cev/ecv/software/SpreaD3_tutorial. 10.1093/bioinformatics/btr481 PMC3187652 21911333

[35] Minin VN, Suchard MA. Counting labeled transitions in continuous-time Markov models of evolution. J Math Biol. 2008;56(3):391–412. 10.1007/s00285-007-0120-8 . 17874105

[36] Lemey P, Rambaut A, Bedford T, Faria N, Bielejec F, Baele G, et al Unifying viral genetics and human transportation data to predict the global transmission dynamics of human influenza H3N2. PLoS Pathog. 2014;10(2):e1003932 10.1371/journal.ppat.1003932 24586153 PMC3930559

[37] Dellicour S, Vrancken B, Trovao NS, Fargette D, Lemey P. On the importance of negative controls in viral landscape phylogeography. Virus Evol. 2018;4(2):vey023 10.1093/ve/vey023 30151241 PMC6101606

[38] Yang Q, Zhao X, Lemey P, Suchard MA, Bi Y, Shi W, et al Assessing the role of live poultry trade in community-structured transmission of avian influenza in China. Proceedings of the National Academy of Sciences. 2020;117(11):5949–54. 10.1073/pnas.1906954117 32123088 PMC7084072

[39] Chong YL, Padhi A, Hudson PJ, Poss M. The effect of vaccination on the evolution and population dynamics of avian paramyxovirus-1. PLoS Pathog. 2010;6(4):e1000872 Epub 2010/04/28. 10.1371/journal.ppat.1000872 20421950 PMC2858710

[40] Abolnik C, Horner RF, Bisschop SP, Parker ME, Romito M, Viljoen GJ. A phylogenetic study of South African Newcastle disease virus strains isolated between 1990 and 2002 suggests epidemiological origins in the Far East. Archives of Virology. 2004;149(3):603–19. 10.1007/s00705-003-0218-2 14991446

[41] Lomniczi B,., Wehmann E,., Herczeg J,., Ballagi-Pordány A,., Kaleta EF, Werner O,., et al Newcastle disease outbreaks in recent years in western Europe were caused by an old (VI) and a novel genotype (VII). Archives of Virology. 1998;143(1):49–64. 10.1007/s007050050267 9505965

[42] Herczeg J, Wehmann E, Bragg RR, Dias PMT, Hadjiev G, Werner O, et al Two novel genetic groups (VIIb and VIII) responsible for recent Newcastle disease outbreaks in Southern Africa, one (VIIb) of which reached Southern Europe. Archives of Virology. 144(11):2087–99. 10.1007/s007050050624 10603164

[43] Yu L, Wang Z, Jiang Y, Chang L, Kwang J. Characterization of newly emerging Newcastle disease virus isolates from the People's Republic of China and Taiwan. Journal of clinical microbiology. 2001;39(10):3512–9. Epub 2001/09/28. 10.1128/JCM.39.10.3512-3519.2001 11574565 PMC88381

[44] Liu XF, Wan HQ, Ni XX, Wu YT, Liu WB. Pathotypical and genotypical characterization of strains of Newcastle disease virus isolated from outbreaks in chicken and goose flocks in some regions of China during 1985–2001. Arch Virol. 2003;148(7):1387–403. 10.1007/s00705-003-0014-z . 12827467

[45] Liu H, Wang J, Ge S, Lv Y, Li Y, Zheng D, et al Molecular characterization of new emerging sub-genotype VIIh Newcastle disease viruses in China. Virus Genes. 2019;55(3):314–21. 10.1007/s11262-019-01651-5 . 30835036

[46] Zhu J, Xu H, Liu J, Zhao Z, Hu S, Wang X, et al Surveillance of avirulent Newcastle disease viruses at live bird markets in Eastern China during 2008–2012 reveals a new sub-genotype of class I virus. Virology journal. 2014;11:211 Epub 2014/12/05. 10.1186/s12985-014-0211-2 25471313 PMC4261539

[47] Lu R. Building Engines for Growth and Competitiveness in China: Experience with Special Economic Zones and Industrial Clusters. Regional Studies. 2011;45(9).

[48] Meng W, Yang Q, Vrancken B, Chen Z, Liu D, Chen L, et al New evidence for the east-west spread of the highly pathogenic avian influenza H5N1 virus between Central Asian and east Asian-Australasian flyways in China. Emerging microbes & infections. 2019;8(1):823–6. Epub 2019/06/06. 10.1080/22221751.2019.1623719 31164049 PMC6567254

[49] Olsen B. Global Patterns of Influenza A Virus in Wild Birds. Pigs & Poultry. 312(5772):384–8. 10.1126/science.1122438 16627734

[50] Hill SC, Lee YJ, Song BM, Kang HM, Lee EK, Hanna A, et al Wild waterfowl migration and domestic duck density shape the epidemiology of highly pathogenic H5N8 influenza in the Republic of Korea. Infection Genetics & Evolution. 2015;34:267–77. 10.1016/j.meegid.2015.06.014 PMC4539883 26079277

[51] Fusaro A, Zecchin B, Vrancken B, Abolnik C, Ademun R, Alassane A, et al Disentangling the role of Africa in the global spread of H5 highly pathogenic avian influenza. Nat Commun. 2019;10(1):5310 10.1038/s41467-019-13287-y 31757953 PMC6874648

[52] Baele G, Dellicour S, Suchard MA, Lemey P, Vrancken B. Recent advances in computational phylodynamics. Current opinion in virology. 2018;31:24–32. 10.1016/j.coviro.2018.08.009 . 30248578

[53] Michael F, Hao H. Extracting transmission networks from phylogeographic data for epidemic and endemic diseases: Ebola virus in Sierra Leone, 2009 H1N1 pandemic influenza and polio in Nigeria. International Health. 2015;7(2):130–8. 10.1093/inthealth/ihv012 25733563 PMC4379986

[54] Magee D, Suchard MA, Scotch M. Bayesian phylogeography of influenza A/H3N2 for the 2014–15 season in the United States using three frameworks of ancestral state reconstruction. Plos Computational Biology. 2017;13(2):e1005389 10.1371/journal.pcbi.1005389 28170397 PMC5321473

[55] Cuypers L, Vrancken B, Fabeni L, Marascio N, Cento V, Di Maio VC, et al Implications of hepatitis C virus subtype 1a migration patterns for virus genetic sequencing policies in Italy. BMC Evol Biol. 2017;17(1):70 10.1186/s12862-017-0913-3 28270091 PMC5341469

