# Supplementary figures and images for: Evolutionary dynamics and transmission patterns of Newcastle disease virus in China through Bayesian phylogeographical analysis
Source: PLoS One. 2020 Sep 29;15(9):e0239809. doi: 10.1371/journal.pone.0239809 (PMC7523974; doi:10.1371/journal.pone.0239809)

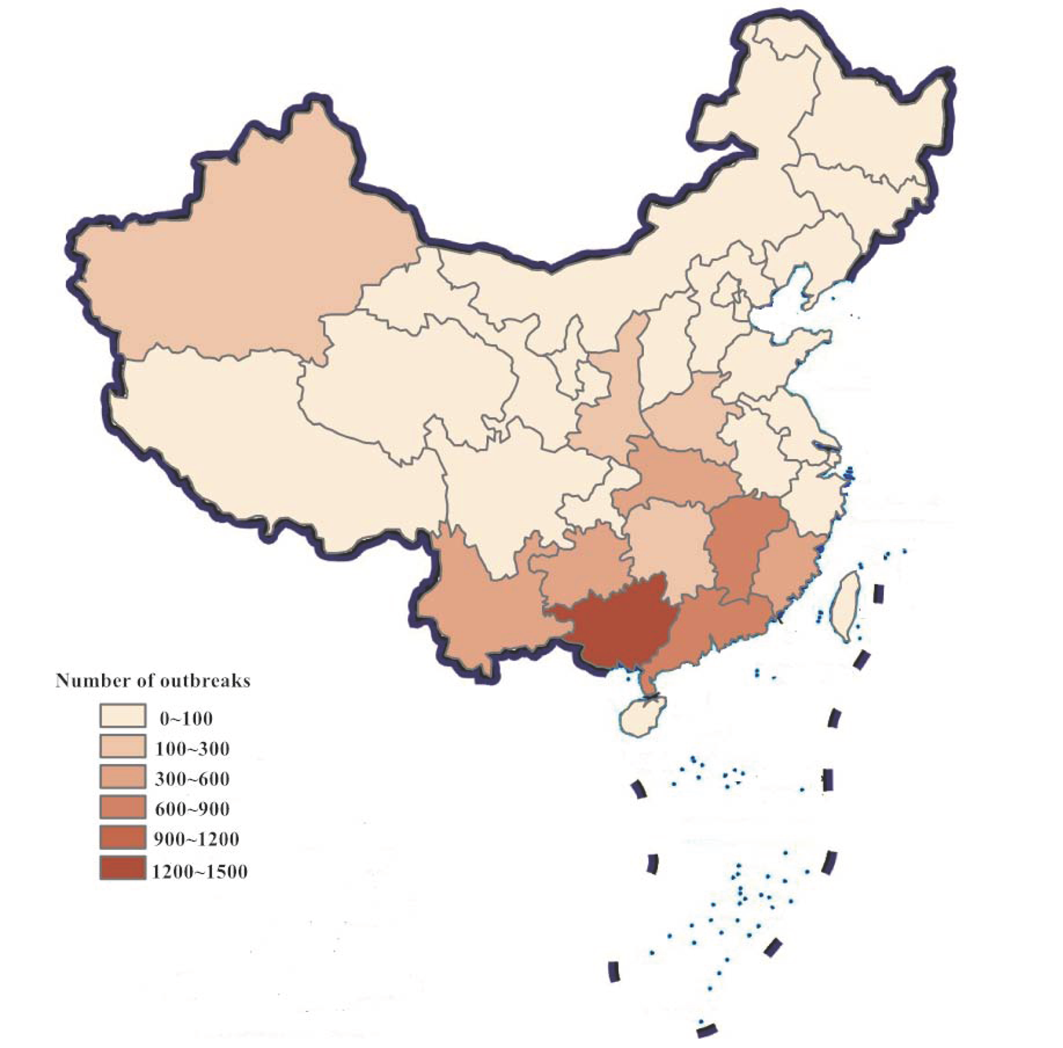

Supplement: S1 Fig — Different shades of color represent the total number of outbreaks in the region. The deeper the color, the more outbreaks of NDV in the region. (TIF) [file pone.0239809.s001.tif]

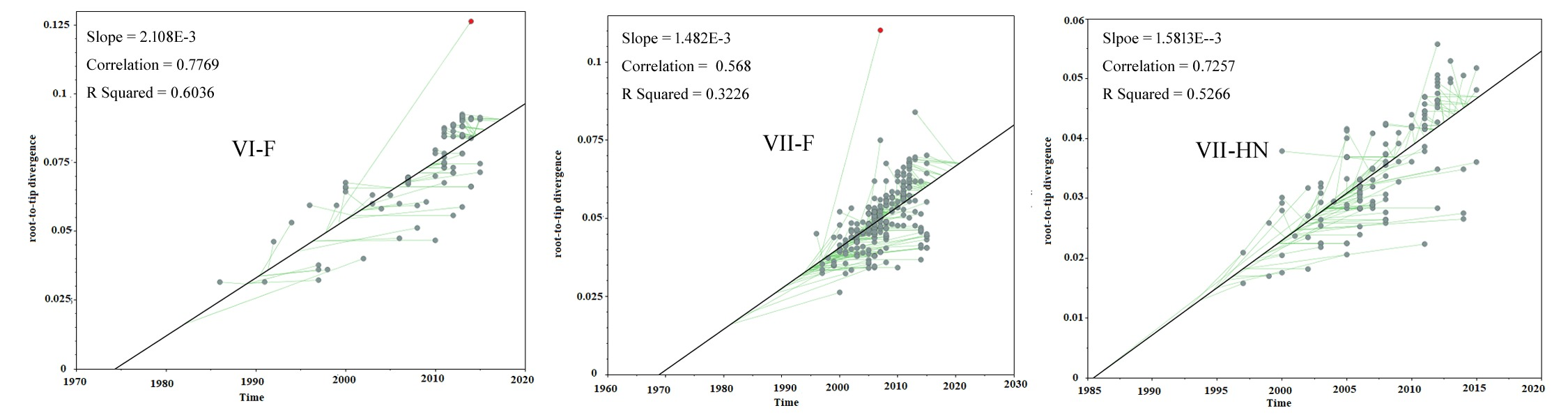

Supplement: S2 Fig — The X-axis represents time, Y-axis represents root-to-tip divergence, the line is the best-fit regression. The red spot represents the problematic sequences and has been removed in the phylogenetic and phylogeographic analysis. (TIF) [file pone.0239809.s002.tif]

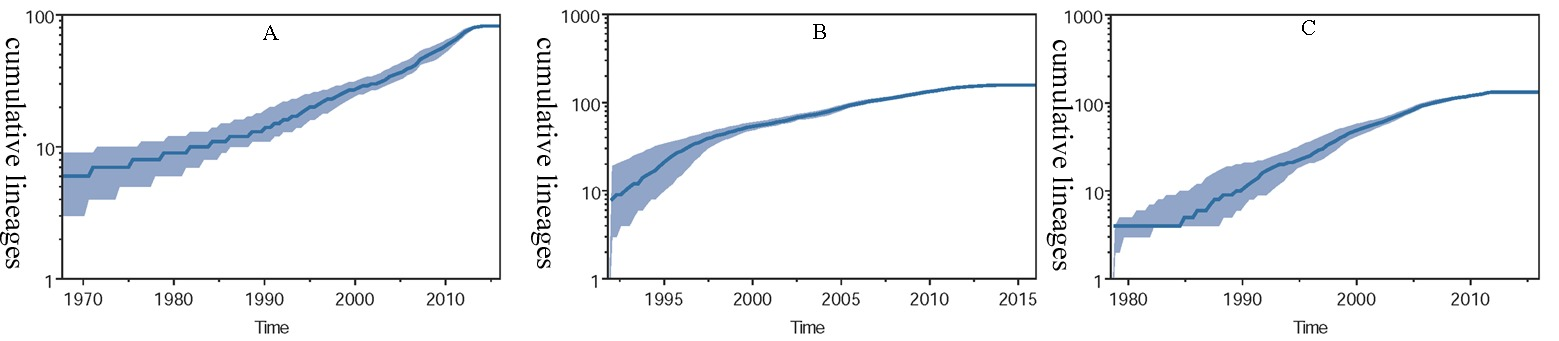

Supplement: S3 Fig — (A) VI-F gene, (B) VII-F gene, (C) VII-HN gene. (TIF) [file pone.0239809.s003.tif]

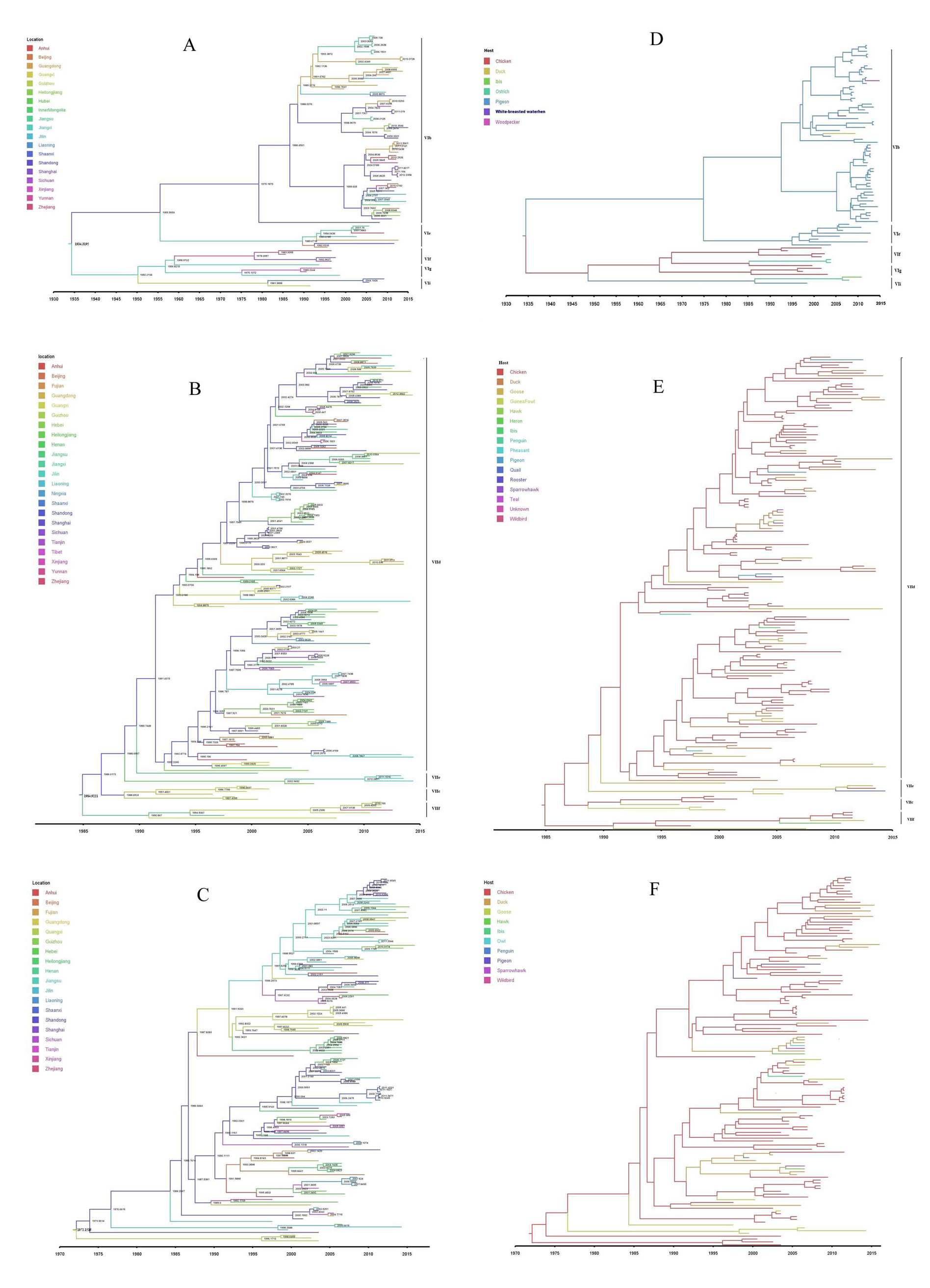

Supplement: S4 Fig — (A), (B) and (C) represent the MCC trees based on locations of VI-F gene, VII-F (subsample 1) and VII-HN genes, respectively; (D), (E) and (F) represent the MCC trees based on hosts of VI-F gene, VII-F (subsample 1) and VII-HN gene respectively. Lines of diverse colors represent different locations or host origins. The scale bar represents the unit of time (year). (TIF) [file pone.0239809.s004.tif]

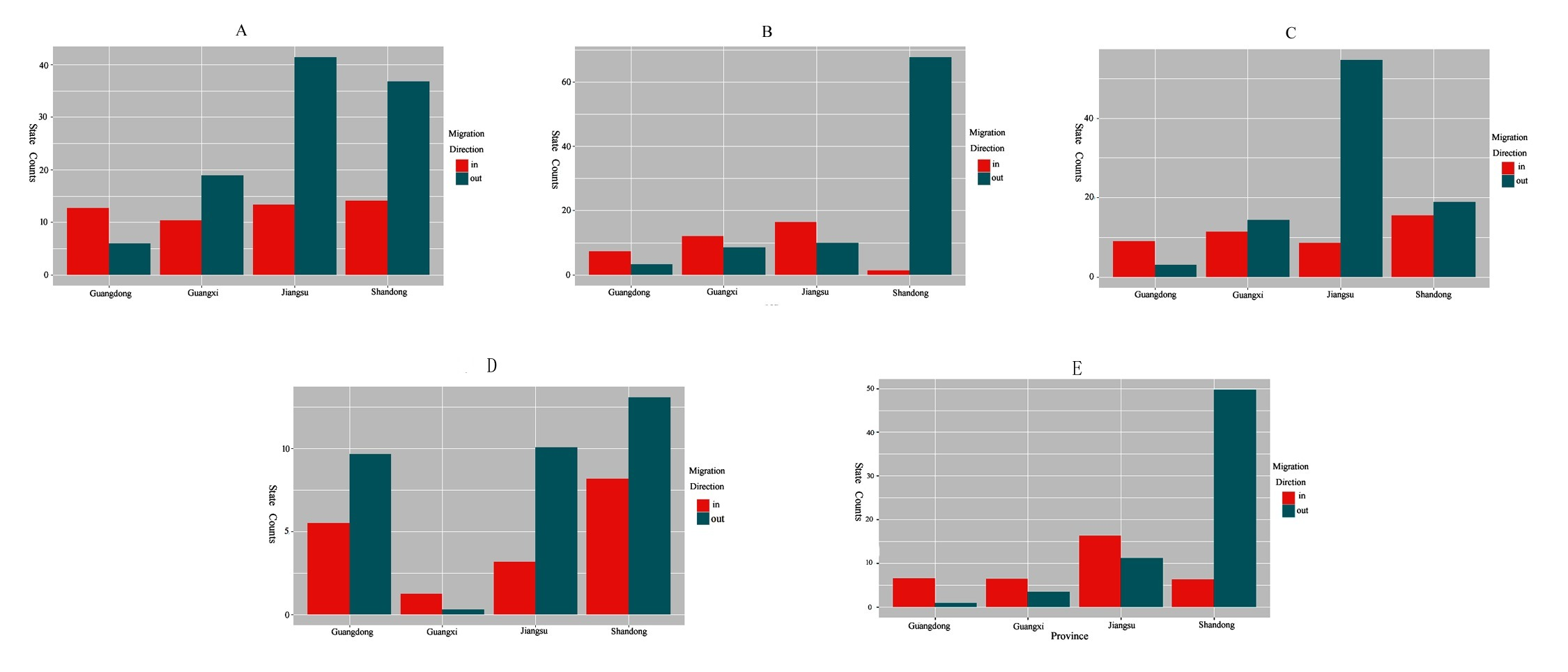

Supplement: S5 Fig — (A) VII-F gene (subsample 1), (B) VII-F gene (subsample 2), (C) VII-F gene (subsample 3), (D) VI-F gene, (E) VII-HN gene. (TIF) [file pone.0239809.s005.tif]

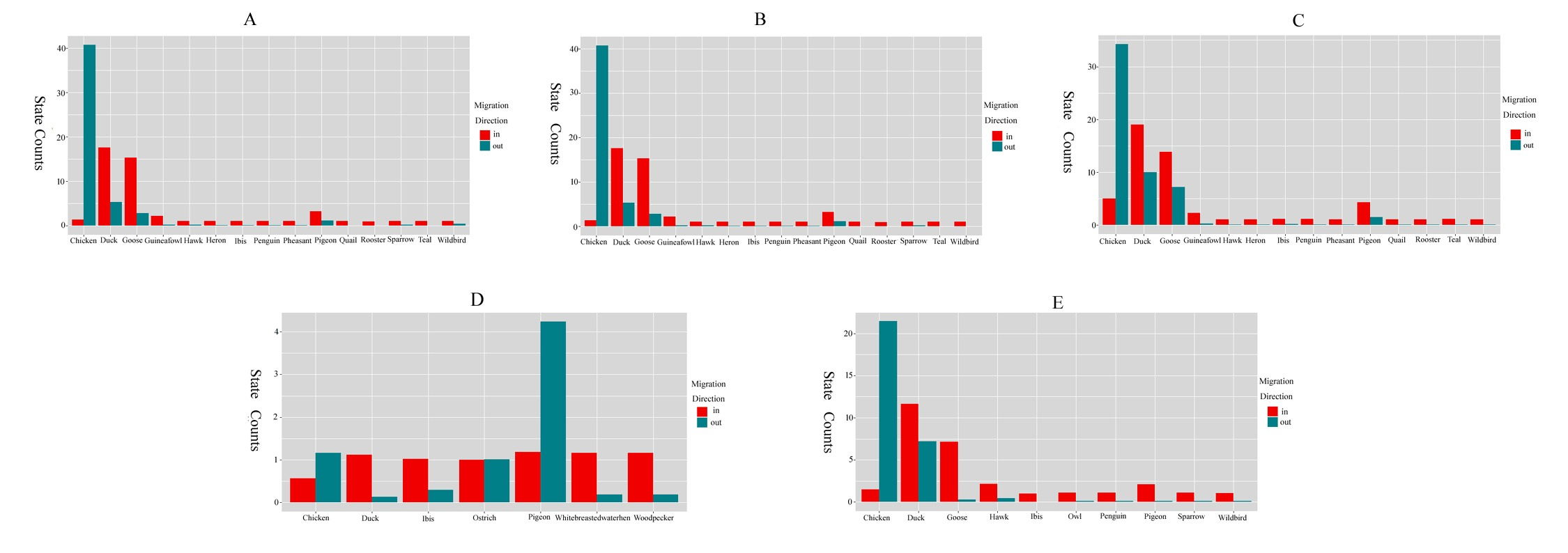

Supplement: S6 Fig — (A) VII-F gene (subsample 1), (B) VII-F gene (subsample 2), (C) VII-F gene (subsample 3), (D) VI-F gene, (E) VII-HN gene. (TIF) [file pone.0239809.s006.tif]
