# Supplementary material for: Evolutionary dynamics and transmission patterns of Newcastle disease virus in China through Bayesian phylogeographical analysis
Source: PLoS One. 2020 Sep 29;15(9):e0239809. doi: 10.1371/journal.pone.0239809 (PMC7523974; doi:10.1371/journal.pone.0239809)
Supplement: S2 Table — (DOCX) [file pone.0239809.s008.docx]

**S2 Table. Best model screening of NDV**

| **Genotype** | **substitution model** | **Clock Model** | **Tree Prior** | **MLE** |
| --- | --- | --- | --- | --- |
| **VI** | GTR+I+G4 | UCLD | Constant Size | -9052.4 |
|  | GTR+I+G4 | UCLD | Exponential Growth | -9031.6 |
|  | GTR+I+G4 | UCLD | Bayesian Skyline | -9014.3 |
|  | GTR+I+G4 | UCED | Constant Size | -8846.9 |
|  | GTR+I+G4 | UCED | Exponential Growth | -8976.4 |
|  | **GTR+I+G4** | **UCED** | **Bayesian Skyline** | **-8842.6** |
|  | GTR+I+G4 | Strict Clock | Constant Size | -9153.2 |
|  | GTR+I+G4 | Strict Clock | Exponential Growth | -9122.9 |
|  | GTR+I+G4 | Strict Clock | Bayesian Skyline | -8996.1 |
| **Ⅶ** | GTR+I+G4 | UCLD | Constant Size | -18792.1 |
|  | GTR+I+G4 | UCLD | Exponential Growth | -18775.3 |
|  | GTR+I+G4 | UCLD | Bayesian Skyline | -18763.2 |
|  | GTR+I+G4 | UCED | Constant Size | -18758.1 |
|  | GTR+I+G4 | UCED | Exponential Growth | -18740.7 |
|  | **GTR+I+G4** | **UCED** | **Bayesian Skyline** | **-18714.9** |
|  | GTR+I+G4 | Strict Clock | Constant Size | -18802.3 |
|  | GTR+I+G4 | Strict Clock | Exponential Growth | -18796.6 |
|  | GTR+I+G4 | Strict Clock | Bayesian Skyline | -18782.4 |

Note: The molecular clock model with higher value of Marginal L estimate was considered the best fit model (black bold). UCLD represents the Relaxed clock with uncorrelated lognormal distribution,

UCED represents relaxed clock with uncorrelated exponential distribution.
