# Supplementary material for: Evolutionary dynamics and transmission patterns of Newcastle disease virus in China through Bayesian phylogeographical analysis
Source: PLoS One. 2020 Sep 29;15(9):e0239809. doi: 10.1371/journal.pone.0239809 (PMC7523974; doi:10.1371/journal.pone.0239809)
Supplement: S5 Table — (DOCX) [file pone.0239809.s011.docx]

**S5 Table. Posterior probabilities and Bayes factor support for diffusion between discrete locations of NDV.**

| **From location A** | **To Location B** | **Bayes factor** | **Posterior probability** |
| --- | --- | --- | --- |
| **VI-F gene** | | | |
| Shandong | Heilongjiang | 334.914 | 0.950894 |
| Heilongjiang | Shaanxi | 157.4257 | 0.901011 |
| Shandong | Beijing | 144.6989 | 0.893234 |
| Guangdong | Guangxi | 60.19411 | 0.776803 |
| Jiangsu | Anhui | 38.34304 | 0.689146 |
| Guangdong | Sichuan | 29.03684 | 0.626708 |
| Jilin | Shaanxi | 26.17746 | 0.602155 |
| Jiangsu | Guangdong | 24.62067 | 0.587379 |
| Shandong | Jilin | 23.94335 | 0.580602 |
| Beijing | Zhejiang | 21.98703 | 0.559716 |
| Guangdong | Jilin | 21.62367 | 0.555605 |
| Zhejiang | Shanghai | 20.53601 | 0.542829 |
| Jiangsu | Shandong | 20.18083 | 0.538496 |
| Guangdong | Hubei | 20.03704 | 0.536718 |
| Jiangxi | Xinjiang | 16.03284 | 0.481058 |
| Shandong | Inner Mongolia | 14.77634 | 0.460727 |
| Guangdong | Shandong | 13.47065 | 0.43784 |
| Xinjiang | Jiangxi | 11.727 | 0.404066 |
| Shaanxi | Guizhou | 11.68378 | 0.403177 |
| Jilin | Liaoning | 10.61856 | 0.380402 |
| Shandong | Zhejiang | 10.35087 | 0.374403 |
| Shandong | Liaoning | 9.896939 | 0.36396 |
| Guangdong | Jiangsu | 8.347182 | 0.325519 |
| Anhui | Yunnan | 8.229458 | 0.322409 |
| Shandong | Shanghai | 7.251445 | 0.295412 |
| Guizhou | Shaanxi | 6.8629 | 0.28408 |
| Beijing | Jiangsu | 5.279434 | 0.233863 |
| Shandong | Yunnan | 5.263077 | 0.233307 |
| Shandong | Guangdong | 4.979038 | 0.223531 |
| Shanghai | Zhejiang | 4.386499 | 0.202311 |
| Jilin | Inner Mongolia | 4.165315 | 0.19409 |
| Liaoning | Jilin | 4.162357 | 0.193978 |
| Shandong | Anhui | 4.085732 | 0.19109 |
| Guangdong | Anhui | 4.068127 | 0.190423 |
| Zhejiang | Jiangxi | 3.960201 | 0.186313 |
| Guangdong | Shanghai | 3.603514 | 0.172425 |
| Jiangsu | Sichuan | 3.414458 | 0.164871 |
| Yunnan | Shanghai | 3.193676 | 0.155872 |
| Guangdong | Yunnan | 3.166745 | 0.154761 |
| Liaoning | Inner Mongolia | 3.147935 | 0.153983 |
| Shanghai | Yunnan | 3.091713 | 0.15165 |
| Jiangsu | Shanghai | 3.086375 | 0.151428 |
| Jiangsu | Yunnan | 3.041112 | 0.149539 |
| **VII-F gene (Subsample 1)** | | | |
| Jilin | Heilongjiang | 972.0953 | 0.978561 |
| Liaoning | Tibet | 434.772 | 0.953302 |
| Guangxi | Shanghai | 364.5292 | 0.9448 |
| Heilongjiang | Beijing | 174.2346 | 0.891079 |
| Jiangsu | Guizhou | 90.57977 | 0.809635 |
| Guizhou | Yunnan | 90.21887 | 0.809019 |
| Shandong | Henan | 82.51654 | 0.79485 |
| Guangxi | Guangdong | 75.91869 | 0.780927 |
| Shandong | Tianjin | 50.42466 | 0.703056 |
| Jiangsu | Shandong | 36.512 | 0.631592 |
| Guangxi | Guizhou | 36.08975 | 0.628881 |
| Jiangsu | Hebei | 32.58355 | 0.604731 |
| Jiangsu | Guangdong | 30.16139 | 0.586126 |
| Shandong | Liaoning | 26.9174 | 0.55828 |
| Hebei | Jilin | 25.48205 | 0.544726 |
| Shandong | Jiangsu | 24.71007 | 0.537087 |
| Guangdong | Fujian | 24.49069 | 0.534869 |
| Liaoning | Tianjin | 22.6066 | 0.514909 |
| Jiangsu | Anhui | 22.51756 | 0.513923 |
| Jiangsu | Jilin | 22.08865 | 0.509118 |
| Shandong | Sichuan | 22.06688 | 0.508871 |
| Guangdong | Zhejiang | 21.86116 | 0.50653 |
| Zhejiang | Tianjin | 19.31585 | 0.475604 |
| Shandong | Jilin | 18.64945 | 0.466856 |
| Shanghai | Jilin | 18.21117 | 0.460941 |
| Jiangsu | Liaoning | 17.5017 | 0.451084 |
| Liaoning | Shandong | 16.83408 | 0.441474 |
| Shandong | Guangdong | 16.72503 | 0.439872 |
| Liaoning | Jiangxi | 16.14844 | 0.431247 |
| Jilin | Guangxi | 16.05941 | 0.429892 |
| Shandong | Anhui | 15.71544 | 0.424593 |
| Jiangsu | Shaanxi | 14.26112 | 0.40106 |
| Jiangsu | Beijing | 13.79904 | 0.393174 |
| Shandong | Shaanxi | 13.01184 | 0.379251 |
| Jiangsu | Guangxi | 12.52842 | 0.370379 |
| Shandong | Hebei | 12.18119 | 0.363849 |
| Tianjin | Hebei | 11.80927 | 0.356703 |
| Hebei | Beijing | 11.52013 | 0.351035 |
| Jiangsu | Heilongjiang | 11.3281 | 0.347215 |
| Shandong | Heilongjiang | 11.30964 | 0.346846 |
| Shandong | Beijing | 10.67632 | 0.333908 |
| Jiangsu | Ningxia | 10.54673 | 0.331198 |
| Jilin | Guizhou | 10.535 | 0.330951 |
| Zhejiang | Hebei | 10.27913 | 0.32553 |
| Guangxi | Jilin | 10.2273 | 0.324421 |
| Beijing | Fujian | 10.1871 | 0.323558 |
| Shandong | Guangxi | 10.13557 | 0.322449 |
| Guangdong | Guangxi | 9.552127 | 0.309635 |
| Fujian | Guangdong | 8.968734 | 0.296328 |
| Jiangsu | Tianjin | 8.831568 | 0.293125 |
| Shaanxi | Heilongjiang | 8.143901 | 0.276614 |
| Guangdong | Guizhou | 7.969426 | 0.272302 |
| Jiangsu | Henan | 7.831328 | 0.268852 |
| Jiangsu | Sichuan | 7.113162 | 0.25037 |
| Jiangsu | Xinjiang | 6.996897 | 0.247289 |
| Tianjin | Zhejiang | 6.886174 | 0.244332 |
| Guangxi | Shandong | 5.643036 | 0.209463 |
| Heilongjiang | Shaanxi | 5.426445 | 0.203056 |
| Tianjin | Sichuan | 5.294881 | 0.199113 |
| Guangxi | Jiangsu | 4.872141 | 0.186175 |
| Shandong | Xinjiang | 4.745966 | 0.182233 |
| Shandong | Ningxia | 4.117951 | 0.162026 |
| Guizhou | Shandong | 3.623274 | 0.145392 |
| Guizhou | Guangxi | 3.48038 | 0.140463 |
| Jiangsu | Jiangxi | 3.462633 | 0.139847 |
| Henan | Shandong | 3.356685 | 0.136151 |
| Guangdong | Ningxia | 3.283059 | 0.133563 |
| Tianjin | Guangxi | 3.237701 | 0.131962 |
| Beijing | Xinjiang | 3.133665 | 0.128265 |
| Beijing | Guangdong | 3.116411 | 0.127649 |
| Shandong | Zhejiang | 3.095739 | 0.12691 |
| **VII-F gene (Subsample 2)** | | | |
| Shandong | Jiangsu | 95838.66 | 1 |
| Shandong | Henan | 3972.867 | 0.994668 |
| Jiangsu | Guizhou | 1119.893 | 0.981337 |
| Shandong | Liaoning | 388.3605 | 0.948012 |
| Shandong | Hebei | 211.3723 | 0.908465 |
| Liaoning | Tibet | 195.5803 | 0.9018 |
| Shandong | Anhui | 190.7821 | 0.899578 |
| Shandong | Tianjin | 172.359 | 0.890024 |
| Hebei | Jilin | 137.6743 | 0.86603 |
| Guizhou | Yunnan | 118.6441 | 0.847812 |
| Shandong | Guangdong | 81.11701 | 0.792046 |
| Liaoning | Tianjin | 62.42301 | 0.745612 |
| Shandong | Shaanxi | 60.56414 | 0.739836 |
| Shandong | Beijing | 45.97267 | 0.683404 |
| Guangxi | Shanghai | 40.23009 | 0.653855 |
| Guizhou | Guangxi | 39.335 | 0.648745 |
| Shanghai | Guangxi | 31.31501 | 0.595201 |
| Shandong | Sichuan | 27.81111 | 0.566319 |
| Guangxi | Jilin | 27.51105 | 0.563653 |
| Shandong | Zhejiang | 27.19011 | 0.560764 |
| Guangdong | Guizhou | 26.41778 | 0.553655 |
| Shandong | Shanghai | 23.74949 | 0.527216 |
| Shanghai | Jilin | 22.07807 | 0.508998 |
| Fujian | Guangdong | 21.9997 | 0.508109 |
| Guangdong | Ningxia | 19.87948 | 0.482782 |
| Shandong | Guangxi | 19.04762 | 0.472117 |
| Heilongjiang | Beijing | 18.77775 | 0.468563 |
| Shaanxi | Heilongjiang | 18.41253 | 0.463675 |
| Beijing | Fujian | 16.81781 | 0.441235 |
| Hebei | Beijing | 14.94447 | 0.412353 |
| Shandong | Jiangxi | 12.3258 | 0.366585 |
| Shandong | Xinjiang | 11.70078 | 0.354588 |
| Guangxi | Guizhou | 10.6985 | 0.33437 |
| Liaoning | Zhejiang | 9.397351 | 0.306154 |
| Jiangsu | Jiangxi | 9.270109 | 0.303266 |
| Heilongjiang | Jilin | 9.124595 | 0.299933 |
| Jilin | Heilongjiang | 7.990622 | 0.272828 |
| Zhejiang | Tianjin | 7.919209 | 0.271051 |
| Beijing | Heilongjiang | 7.901411 | 0.270607 |
| Tianjin | Zhejiang | 7.593405 | 0.26283 |
| Tianjin | Hebei | 7.420302 | 0.258387 |
| Guangdong | Guangxi | 6.375718 | 0.230393 |
| Jiangsu | Guangxi | 5.835256 | 0.215063 |
| Shandong | Heilongjiang | 5.827579 | 0.214841 |
| Liaoning | Jiangsu | 5.728164 | 0.211953 |
| Guangxi | Guangdong | 5.494012 | 0.205066 |
| Henan | Sichuan | 5.42679 | 0.203066 |
| Henan | Shaanxi | 5.241822 | 0.197512 |
| Hebei | Heilongjiang | 5.081154 | 0.192624 |
| Hebei | Anhui | 4.951139 | 0.188625 |
| Henan | Guangxi | 4.737272 | 0.18196 |
| Zhejiang | Hebei | 4.37471 | 0.170407 |
| Zhejiang | Jiangsu | 4.176864 | 0.163964 |
| Shandong | Jilin | 4.149814 | 0.163075 |
| Liaoning | Shandong | 4.075722 | 0.160631 |
| Liaoning | Jiangxi | 3.982045 | 0.157521 |
| Shandong | Ningxia | 3.816473 | 0.151966 |
| Guangdong | Fujian | 3.724696 | 0.148856 |
| Shandong | Fujian | 3.60121 | 0.144635 |
| Jiangsu | Guangdong | 3.294608 | 0.13397 |
| Jiangxi | Xinjiang | 3.088162 | 0.126639 |
| Heilongjiang | Shaanxi | 3.057184 | 0.125528 |
| Henan | Ningxia | 3.044815 | 0.125083 |
| Zhejiang | Shandong | 3.020115 | 0.124195 |
| Beijing | Xinjiang | 3.020115 | 0.124195 |
| **VII-F gene (Subsample 3)** | | | |
| Jiangsu | Shandong | 19148.56 | 0.998889 |
| Jilin | Heilongjiang | 3595.658 | 0.994112 |
| Jiangsu | Henan | 1328.693 | 0.984224 |
| Jiangsu | Liaoning | 599.0864 | 0.96567 |
| Jiangsu | Hebei | 288.8945 | 0.931341 |
| Guangxi | Shanghai | 282.5038 | 0.929897 |
| Liaoning | Tibet | 225.4189 | 0.913676 |
| Shandong | Guizhou | 154.4116 | 0.878791 |
| Guangxi | Guangdong | 142.1285 | 0.869681 |
| Jiangsu | Anhui | 120.8068 | 0.850128 |
| Guizhou | Yunnan | 117.1131 | 0.846128 |
| Liaoning | Zhejiang | 101.5075 | 0.826575 |
| Jiangsu | Shaanxi | 89.63922 | 0.808021 |
| Shandong | Guangxi | 67.08363 | 0.759027 |
| Henan | Sichuan | 54.92463 | 0.720587 |
| Shanghai | Jilin | 54.71307 | 0.719809 |
| Liaoning | Tianjin | 54.41287 | 0.718698 |
| Shandong | Jiangsu | 47.68367 | 0.691257 |
| Jiangsu | Jilin | 44.71444 | 0.677369 |
| Jiangsu | Beijing | 43.64108 | 0.672036 |
| Heilongjiang | Beijing | 34.96862 | 0.621487 |
| Guangdong | Shandong | 27.34465 | 0.56216 |
| Zhejiang | Tianjin | 18.16292 | 0.460282 |
| Jiangsu | Jiangxi | 17.91271 | 0.456838 |
| Anhui | Shandong | 17.43734 | 0.450172 |
| Jiangsu | Xinjiang | 14.36738 | 0.402844 |
| Hebei | Beijing | 13.72236 | 0.391845 |
| Guangxi | Guizhou | 13.51251 | 0.388179 |
| Liaoning | Jiangxi | 12.92219 | 0.377625 |
| Jiangsu | Tianjin | 12.60748 | 0.371848 |
| Anhui | Guangdong | 12.35747 | 0.367181 |
| Tianjin | Hebei | 12.1987 | 0.364182 |
| Shaanxi | Heilongjiang | 12.1987 | 0.364182 |
| Jiangsu | Guangdong | 11.97775 | 0.35996 |
| Beijing | Fujian | 11.77111 | 0.35596 |
| Henan | Guangxi | 11.75971 | 0.355738 |
| Shandong | Guangdong | 10.65229 | 0.333407 |
| Henan | Tianjin | 10.53028 | 0.330852 |
| Jiangsu | Guangxi | 8.905548 | 0.294856 |
| Tianjin | Henan | 8.735203 | 0.290857 |
| Zhejiang | Hebei | 8.322208 | 0.280969 |
| Guangxi | Jiangsu | 8.253717 | 0.279302 |
| Tianjin | Sichuan | 7.92487 | 0.271192 |
| Tianjin | Guangxi | 7.871512 | 0.269859 |
| Jiangsu | Ningxia | 7.703824 | 0.265637 |
| Jiangsu | Fujian | 7.699437 | 0.265526 |
| Fujian | Guangdong | 7.681903 | 0.265082 |
| Guangxi | Jilin | 7.629428 | 0.263748 |
| Beijing | Heilongjiang | 7.378454 | 0.257305 |
| Jiangsu | Zhejiang | 6.488955 | 0.23353 |
| Liaoning | Xinjiang | 5.151005 | 0.194756 |
| Guangdong | Ningxia | 4.915923 | 0.187535 |
| Heilongjiang | Shaanxi | 4.614742 | 0.178091 |
| Tianjin | Zhejiang | 4.113259 | 0.161871 |
| Shanghai | Guangxi | 3.969242 | 0.157094 |
| Anhui | Fujian | 3.695821 | 0.147872 |
| Jiangsu | Sichuan | 3.617859 | 0.145206 |
| Guizhou | Guangxi | 3.488998 | 0.140762 |
| Jilin | Jiangsu | 3.428254 | 0.138651 |
| Jiangsu | Heilongjiang | 3.425065 | 0.13854 |
| Anhui | Ningxia | 3.207009 | 0.130874 |
| Fujian | Shandong | 3.200746 | 0.130652 |
| Guangdong | Guizhou | 3.169478 | 0.129541 |
| **VII-HN gene** | | | |
| Shandong | Jiangsu | 3034.723 | 0.994333 |
| Shandong | Henan | 487.2721 | 0.965722 |
| Jiangsu | Guizhou | 421.7668 | 0.960608 |
| Liaoning | Jilin | 370.1124 | 0.955356 |
| Shandong | Heilongjiang | 260.7773 | 0.937802 |
| Tianjin | Liaoning | 226.1535 | 0.928956 |
| Guangxi | Guangdong | 215.7264 | 0.925777 |
| Shandong | Tianjin | 206.1559 | 0.922598 |
| Shandong | Anhui | 183.2378 | 0.913753 |
| Tianjin | Zhejiang | 84.52125 | 0.830131 |
| Tianjin | Hebei | 81.23426 | 0.824464 |
| Tianjin | Xinjiang | 57.49996 | 0.768763 |
| Shandong | Guangxi | 52.72845 | 0.753006 |
| Jilin | Shandong | 47.70856 | 0.733932 |
| Fujian | Shaanxi | 32.73762 | 0.654319 |
| Anhui | Beijing | 26.59536 | 0.605943 |
| Jiangsu | Guangdong | 25.82402 | 0.598894 |
| Jiangsu | Shaanxi | 23.45111 | 0.575536 |
| Anhui | Fujian | 18.40574 | 0.515549 |
| Shandong | Guangdong | 14.87233 | 0.462336 |
| Beijing | Fujian | 11.05359 | 0.38991 |
| Guangxi | Shanghai | 9.919131 | 0.364478 |
| Heilongjiang | Jilin | 8.676455 | 0.33407 |
| Hebei | Beijing | 8.107034 | 0.319143 |
| Jilin | Heilongjiang | 7.522605 | 0.30311 |
| Xinjiang | Jilin | 7.385543 | 0.29924 |
| Shandong | Hebei | 6.912836 | 0.285556 |
| Heilongjiang | Beijing | 6.694702 | 0.27906 |
| Jiangsu | Shandong | 6.690103 | 0.278922 |
| Shandong | Shaanxi | 5.715331 | 0.248376 |
| Tianjin | Beijing | 5.157918 | 0.229717 |
| Xinjiang | Beijing | 4.942442 | 0.222253 |
| Guangdong | Shanghai | 4.891185 | 0.220456 |
| Shandong | Beijing | 4.808881 | 0.217554 |
| Hebei | Zhejiang | 4.219787 | 0.19613 |
| Jiangsu | Guangxi | 4.179171 | 0.19461 |
| Shandong | Sichuan | 4.058239 | 0.190048 |
| Beijing | Jilin | 3.97114 | 0.186731 |
| Hebei | Tianjin | 3.884749 | 0.183414 |
| Beijing | Anhui | 3.494151 | 0.168072 |
| Guangxi | Sichuan | 3.141037 | 0.153697 |
| Henan | Sichuan | 3.1377 | 0.153559 |
| Tianjin | Guangxi | 3.101063 | 0.152039 |
