# Supplementary material for: Evolutionary dynamics and transmission patterns of Newcastle disease virus in China through Bayesian phylogeographical analysis
Source: PLoS One. 2020 Sep 29;15(9):e0239809. doi: 10.1371/journal.pone.0239809 (PMC7523974; doi:10.1371/journal.pone.0239809)
Supplement: S6 Table — (DOCX) [file pone.0239809.s012.docx]

**S6 Table. Posterior probabilities and Bayes factor support for diffusion between diverse hosts of NDV.**

| **From Host A** | **To Host B** | **Bayes factor** | **Posterior probability** |
| --- | --- | --- | --- |
| **VI-F gene** | | | |
| Pigeon | White-breasted water hen | 124.4543 | 0.959338 |
| Pigeon | Woodpecker | 111.6731 | 0.954894 |
| Pigeon | Duck | 42.34875 | 0.889235 |
| Ostrich | Ibis | 23.44903 | 0.816354 |
| Chicken | Pigeon | 8.017464 | 0.603155 |
| Pigeon | Ostrich | 5.435719 | 0.507499 |
| Pigeon | Chicken | 3.658312 | 0.40951 |
| **VII-F gene (Subsample 1)** | | | |
| Chicken | Duck | 115990.1 | 1 |
| Chicken | Goose | 115990.1 | 1 |
| Chicken | Pigeon | 1059.822 | 0.986693 |
| Chicken | Guinea Fowl | 515.4072 | 0.973016 |
| Duck | Teal | 245.2244 | 0.944924 |
| Duck | Wild bird | 106.6706 | 0.881838 |
| Goose | Chicken | 44.47287 | 0.756777 |
| Duck | Penguin | 42.10165 | 0.74655 |
| Duck | Owl | 39.96508 | 0.73657 |
| Duck | Hawk | 38.72517 | 0.730409 |
| Duck | Rooster | 30.20398 | 0.678783 |
| Goose | Quail | 28.24591 | 0.663997 |
| Chicken | Ibis | 23.53012 | 0.622104 |
| Duck | Mink | 12.72846 | 0.471045 |
| Wild bird | Mink | 9.939919 | 0.410177 |
| Goose | Heron | 7.921258 | 0.35658 |
| Chicken | Pheasant | 5.965942 | 0.29448 |
| Owl | Hawk | 5.345167 | 0.272178 |
| Hawk | Owl | 4.664769 | 0.246057 |
| Chicken | Heron | 4.432192 | 0.236693 |
| Duck | Pheasant | 4.347938 | 0.233243 |
| Pigeon | Pheasant | 3.592056 | 0.200838 |
| Goose | Pheasant | 3.528857 | 0.198004 |
| Duck | Heron | 3.158847 | 0.181 |
| **VII-F gene (Subsample 2)** | | | |
| Chicken | Duck | 64319.85 | 1 |
| Chicken | Goose | 4934.487 | 0.997112 |
| Chicken | Pigeon | 918.0856 | 0.98467 |
| Duck | Teal | 492.2747 | 0.971784 |
| Chicken | Guinea Fowl | 403.4609 | 0.965785 |
| Duck | Wild bird | 161.483 | 0.918685 |
| Pigeon | Rooster | 138.1573 | 0.906243 |
| Goose | Quail | 54.2932 | 0.791602 |
| Duck | Hawk | 46.00112 | 0.762942 |
| Duck | Sparrow | 43.19923 | 0.751389 |
| Duck | Penguin | 42.14016 | 0.746723 |
| Chicken | Unknow | 34.44469 | 0.706732 |
| Chicken | Ibis | 33.22084 | 0.699178 |
| Goose | Chicken | 18.06794 | 0.55832 |
| Chicken | Pheasant | 15.92471 | 0.526994 |
| Goose | Heron | 5.969422 | 0.294601 |
| Chicken | Heron | 5.893127 | 0.291935 |
| Hawk | Sparrow | 5.255209 | 0.268829 |
| Sparrow | Hawk | 5.061135 | 0.261497 |
| Duck | Heron | 3.193556 | 0.182626 |
| **VII-F gene (Subsample 3)** | | | |
| Chicken | Duck | 110621.9 | 1 |
| Duck | Goose | 499.9039 | 0.976003 |
| Chicken | Guinea Fowl | 377.2656 | 0.968448 |
| Chicken | Goose | 184.217 | 0.937451 |
| Duck | Teal | 182.4871 | 0.936896 |
| Duck | Wild bird | 66.62036 | 0.84424 |
| Duck | Penguin | 51.10935 | 0.806133 |
| Chicken | Ibis | 38.80991 | 0.759471 |
| Goose | Chicken | 31.98006 | 0.722364 |
| Goose | Pigeon | 30.34227 | 0.711699 |
| Chicken | Pheasant | 11.55225 | 0.484502 |
| Goose | Quail | 10.24569 | 0.454616 |
| Duck | Rooster | 9.384654 | 0.432952 |
| Pigeon | Rooster | 7.908456 | 0.391512 |
| Goose | Heron | 7.592607 | 0.381846 |
| Duck | Hawk | 7.408497 | 0.376069 |
| Pigeon | Quail | 6.120091 | 0.332408 |
| Pigeon | Chicken | 6.049889 | 0.329852 |
| Chicken | Pigeon | 5.99532 | 0.327852 |
| Duck | Pigeon | 4.909266 | 0.285413 |
| Chicken | Heron | 4.494323 | 0.267748 |
| Chicken | Hawk | 4.3479 | 0.261304 |
| Duck | Chicken | 4.060168 | 0.248306 |
| Duck | Pheasant | 3.153908 | 0.2042 |
| **VII-HN gene** | | | |
| Chicken | Duck | 59933.04 | 1 |
| Chicken | Goose | 59933.04 | 0.999862 |
| Duck | Hawk | 573.6697 | 0.985764 |
| Chicken | Pigeon | 302.2919 | 0.973324 |
| Duck | Owl | 128.8806 | 0.939599 |
| Duck | Sparrow | 86.70926 | 0.912785 |
| Duck | Penguin | 69.6622 | 0.893711 |
| Duck | Chicken | 16.91111 | 0.671182 |
| Duck | Wild bird | 15.02949 | 0.644644 |
| Chicken | Ibis | 12.51361 | 0.601659 |
| Hawk | Wild bird | 5.406576 | 0.394886 |
| Goose | Ibis | 3.26893 | 0.28293 |

**Note: Hosts of Chicken, Duck, Goose, Pigeon and Guinea Fowl** **are considered to be poultry, other hosts are all considered to be wild birds.**
